# Combining the third molar mineralization to further improve the accuracy of the Kvaal’s method in dental age estimation of subadults in northern China

**DOI:** 10.1093/fsr/owad013

**Published:** 2023-03-28

**Authors:** Weifeng Qu, Jifeng Cai, Bowei Jiang, Dan Wen, Wei He, Chudong Wang, Hao Xing, Zedeng Yang, Jienan Li, Lagabaiyila Zha, Ying Liu, Jian Zhou

**Affiliations:** Department of Forensic Science, School of Basic Medical Sciences, Central South University, Changsha, China; Department of Forensic Science, School of Basic Medical Sciences, Central South University, Changsha, China; Department of Scientific Instrument, The First Research Institute of the Ministry of Public Security of P.R.C, Beijing, China; Department of Forensic Science, School of Basic Medical Sciences, Central South University, Changsha, China; Department of Forensic Science, School of Basic Medical Sciences, Central South University, Changsha, China; Department of Forensic Science, School of Basic Medical Sciences, Central South University, Changsha, China; Department of Forensic Science, School of Basic Medical Sciences, Central South University, Changsha, China; Department of Forensic Science, School of Basic Medical Sciences, Central South University, Changsha, China; Department of Forensic Science, School of Basic Medical Sciences, Central South University, Changsha, China; Department of Forensic Science, School of Basic Medical Sciences, Central South University, Changsha, China; Department of Oral Implantology, Xiangya Hospital of Stomatology, Central South University, Changsha, China; Department of Integrated Emergency Dental Care and General Dentistry, Capital Medical University School of Stomatology, Beijing, China

**Keywords:** forensic sciences, dental age, subadults, deposition of secondary dentin, mineralization of the third molar, northern China

## Abstract

The morphological changes based on deposition of secondary dentin and mineralization of the third molar have been proven to be related to chronological age. However, Kvaal’s method on the theory of deposition of secondary dentin was controversial with respect to dental age estimation in the recent research. The aim of this study was to combine the parameters of Kvaal’s method with relatively high correlation coefficients and mineralization stages of the third molar to improve the accuracy of predicting the dental age of subadults in northern China. A total of 340 digital orthopantomograms of subadults aged from 15 to 21 years were analysed. A training group was used to test the accuracy of the original Kvaal’s method and to establish novel methods for subadults in northern China. A testing group was used to compare the accuracy of the newly established methods with the Kvaal’s original method and with published method specifically used in northern China. To increase the feasibility of our estimation model, we combined the mineralization of the third molar to build a combined specific formula. The results showed that the combined specific model increased the coefficient of determination to 0.513, and the standard error of the estimate was reduced to 1.482 years. We concluded that the combined specific model based on the deposition of secondary dentin and mineralization of the third molar could improve the accuracy of dental age assessment of subadults in northern China.

**Key Points:**

- The decrease in the dental pulp cavity based on deposition of secondary dentin is a useful variable for assessing age.
- A total of 340 orthopantomographs were used in this research, including 278 in training groups and 62 in testing groups.
- Original Kvaal’s method underestimated the dental age for subadults in northern China.
- The equation of combined specific method constructed in our study was proved more suitable to calculate dental age for subadults in northern China.

## Introduction

Age estimation plays an important role in forensic anthropology [1]. The Study Group of Forensic Age Diagnostics claims that three methods can be employed for age estimation: physical examination, radiography of the left hand, and dental examination [2]. Unlike other indicators, teeth are the hardest tissues in body, so they are highly resistant to physical or chemical impact [3]. Previously, forensic scientists were required to estimate dental age (DA) from unidentified cadavers by physical extraction [4]. With the advent of radiographic technology, noninvasive methods of age estimation have become more popular [5]. To adapt to social demands, appropriate methods for estimation of DA must be accurate, noninvasive, and ethically acceptable. In recent years, estimation of DA has garnered considerable attention in criminal conflicts and civil conflicts such as identification of unidentified cadavers in murder cases or investigations of chronological age (CA) for immigration purposes [4, 6, 7]. For example, a number of refugees have been emigrating to European countries, and their CAs have become a major issue. Thanks to protection by the United Nations Children’s Charter, people aged <18 years cannot to be sent back to the country they came from [8].

Deposition of secondary dentin begins when the tooth crown is fully formed, and it has been shown to be correlated with CA*.* In 1995, Kvaal et al*.* [9] described a new method (hereafter, termed as “Kvaal’s method”) for age estimation by observing the periapical dental radiographs based on secondary dentin and by measuring a series of parameters. They documented high correlation between the CA and a reduction in the pulp cavity. In 2005, Paewinsky et al*.* [10] found that digital panoramic radiographs could be used to measure the length and width of teeth, which were important parameters in Kvaal’s method. Subsequently, Kvaal’s method started to be gradually applied worldwide.

In China, people older than 18 years are fully responsible for their behaviour [11]. Estimating DA at this age juncture is a difficult stuff because permanent teeth are, in general, developed. Upon this age range, Demirjian’s third molar developmental stages’ division method attracted researchers’ interest. Based on Demirjian’s method, the third molar mineralization had obvious advantages of simple operation process and relatively high accuracy. When other teeth have erupted and completed the root formation in subadults, mineralization of third molar has been proven to be applicable for age prediction in this population [12, 13].

Therefore, it appears that the decrease in pulp cavity could be a good indicator for age estimation around the age of 18 years. The formation of secondary dentin deposition begins when the apices close; meanwhile, development of third molar was also a good indicator [14]. Considering the previous performance of Kvaal’s method in age estimation [15], it is necessary to evaluate the accuracy of this method in Chinese subadults. Therefore, we aimed to verify the applicability of Kvaal’s method for northern Chinese subadults aged between 15 and 21 years and to establish a new system based on Kvaal’s method and mineralization of the third molar to improve the accuracy of CA estimation.

## Materials and methods

### Samples

A total of 340 orthopantomograms (OPGs) were selected from the Capital Medical University School of Stomatology (Beijing, China) from patients aged between 15 and 21 years (171 males and 169 females) with written approval of usage in the present study. The Ethics Committee of Xiangya Hospital approved this study (No. 2019-S234). Among them, 278 samples were assigned to the training group (Supplementary Table S1) and the remaining 62 samples were assigned to the testing group. The inclusion criteria were: (i) OPGs must contain eight types of teeth (maxillary incisor (termed Teeth 21)), lateral incisor (22), second premolar (25), third molar (28), mandibular lateral incisor (32), canine (33), first premolar (34), and third molar (38)); (ii) individuals born and living in Beijing; and (iii) patients should have normal occlusion. Based on statistical analyses in previous studies [9, 10] that the side of the oral cavity had little influence upon Kvaal’s method, we chose to observe the left side for consistency. The exclusion criteria were: (i) disorders in the oral cavity that could affect observation and measurement (e.g., tooth decay, periodontitis, severe attrition of teeth, impacted teeth, rotated teeth, pathological diseases, and genetic anomalies) and (ii) OPGs of low-image quality.

### Methods

OPGs were provided in JPG formats and measured on Photoshop 7.0 (Adobe, San Jose, CA, USA). Based on the analysis of Kvaal et al*.* [9] that the final results were not influenced by which side of the jaw the teeth were selected from, six types of teeth on the left side–Teeth 21, 22, 25, 32, 33 and 34–were chosen for measurements. OPGs were taken during routine treatment of individuals presenting for clinical evaluation, so unnecessary or repeated exposure to radiation was avoided.

**Figure 1:**
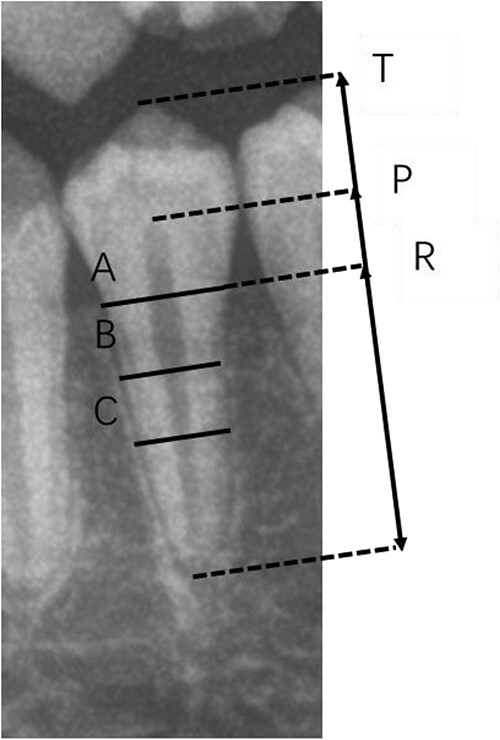
Description of Kvaal’s method. T, maximum tooth length; R, root length on the mesial surface; P, maximum pulp length; A, root and pulp width at enamel–cementum junction (ECJ); B, root and pulp width midway between measurement levels A and C; C, root and pulp width midway between apex and ECJ.

Kvaal’s method is described as Figure 1. We measured the maximum tooth length, pulp length, root length, and the width of the root and pulp at the enamel–cementum junction (ECJ) (level A), halfway between the ECJ and midroot level (level B), and midroot level (level C). Although OPGs can be affected by magnification and photo-angulation, six types of dental ratios may help to reduce these errors: pulp/root length (ratio P), tooth/root length (ratio T), pulp/tooth length (ratio R), and the pulp/root widths at the three levels (ratios A, B, and C). The mean values of different ratios were selected as predictors: “M” represented the mean value of all ratios, “W” referred to the mean value from ratios B and C, and “L” represented the mean value of ratios P and R.

For the mineralization of the third molar (Teeth 28 and 38), the developmental process was divided into nine stages based on Demirjian’s morphological stage division method [16], and one stage was added for the appearance of third molar bud but without calcification, which was developed by other researchers [12, 17].

**Table 1:** Correlation coefficient between chronological age and ratios of measurements from digital panoramic.

Parameters	Teeth						Maxillary	Mandibular	Bimaxillary
	21	22	25	32	33	34			
P	0.121^*^	0.028	0.144^*^	0.085	0.120^*^	0.016	0.143^*^	0.099	0.140^*^
T	0.174^**^	0.098	0.007	−0.008	0.025	0.024	0.147^*^	0.016	0.102
R	−0.077	−0.065	0.120	0.110	0.112	0.001	−0.015	0.104	0.046
A	0.136^*^	0.001	−0.023	0.168^**^	0.056	0.070	0.059	0.090	0.100
B	0.085	0.114	0.113	0.206^**^	−0.008	−0.069	0.136^*^	0.008	0.073
C	0.155^**^	0.102	0.162^**^	0.281^**^	0.069	0.072	0.187^**^	0.199^**^	0.299^**^
M	0.200^**^	0.112	0.137	0.175^**^	0.092	0.008	0.208^**^	0.126^*^	0.188^**^
W	0.131^*^	0.128^*^	0.150^*^	0.267^**^	0.019	−0.034	0.180^**^	0.094	0.158^**^
L	0.062	−0.013	0.166^**^	0.113	0.141^*^	0.012	0.097	0.123^*^	0.126
W–L	0.022	0.106	−0.014	0.109	−0.078	−0.038	0.058	0.015	0.049

P, ratio between length of pulp and root; T, ratio between length of tooth and root; R, ratio between length of pulp and tooth; A, ratio between width of pulp and root at CEJ (level A), B, ratio between width of pulp and root at midpoint between level C and A (level B); C, ratio between width of pulp and root at midroot the level (level C); M, mean value of all ratios except for T; W, mean value of width ratios from levels B and C; L, mean value of the length ratios P and R; W–L, difference between W and L.

^*^ *P* < 0.05.

^**^ *P* < 0.01.

**Table 2:** Kvaal-specific equations for age estimation for subadults in northern China.

Teeth	*n*	*R*	*R* ^2^	Equation	SEE
21	280	0.200	0.040	12.560 + 8.192 (M)	2.021
22	280	0.128	0.016	17.345 + 4.740 (W)	2.046
25	280	0.214	0.046	11.856 + 5.889 (L) +5.428 (C)	2.019
32	280	0.281	0.079	16.413 + 9.903 (C)	1.980
33	280	0.141	0.020	12.604 + 6.006 (L)	2.043
34	280	0.072	0.005	17.866 + 2.977 (C)	2.058
Maxillary		0.208	0.043	8.678 + 13.753 (M)	2.019
Mandibular		0.199	0.040	16.291 + 10.565 (C)	2.022
Bimaxillary		0.229	0.053	15.559 + 13.836 (C)	2.008

M, mean value of all ratios except for T; W, mean value of width ratios from levels B and C; L, mean value of the length ratios P and R; *R*, correlation coefficients; *R*^2^, coefficient of determination; SEE, standard error of the estimate in years.

### Statistical analyses

To test the reliability and feasibility of our measurements, 50 samples were selected randomly and were evaluated twice after 3 months from the first measurement by the same and a second observer. For the data measured by Kvaal’s method, the intraclass correlation coefficient was used to evaluate intra- and inter-rater agreements, and all tests were conducted under similar conditions. Meanwhile, Kappa tests were conducted to evaluate the intra- and inter-rater agreements for stage evaluation. Measurement data were inputted into SPSS v21.0 (IBM, Armonk, NY, USA). Then, a sample of all the ratios was used to create a Kvaal-specific equation in the training group. Finally, we applied the third molar mineralization based on Demirjian’s morphological stage method to the Kvaal-specific equation in the training group samples and verified it in the testing group. At the same time, we added a published Chinese method [18] and a method developed by Demirjian’s stage division method [12] for comparison.

## Results

For Kvaal’s method, the intra- and inter-rater reproducibility were 0.906–0.943 and 0.928–0.956, respectively. For the Demirjian’s stage deviation, the intra- and inter-rater agreements were 0.910 and 0.891, respectively.

The results of age estimation by Kvaal’s method and related statistical parameters are shown in Supplementary Table S2. The lowest mean differences between CA and DA were 14.56 years for males and 5.58 years for females, both in Teeth 34. The lowest standard deviation of DA for males was 6.13 years for Teeth 22 in the age group 16.00–16.99 years, while for females, the lowest standard deviation was 5.98 years for Teeth 33 in the age group 17.00–17.99 years.

The correlation coefficients between the CA and different ratios of measurements and the corresponding mean values are shown in Table 1. Among these ratios, the correlation between CA and the dental ratios was assessed using Pearson’s correlation coefficient. The correlation coefficient of ratio C was relatively high and of significant difference except in Teeth 22, 33, and 34. Compared with the length ratios, the width ratios correlated significantly with age for maxillary, mandibular, and bimaxillary teeth (left maxillary and mandibular teeth), and ratio C value had the strongest correlation with CA for the bimaxillary teeth (*R* = 0.299).

We input all variations to SPSS statistical software as arguments and CA as dependent variable to build mathematical models of DA estimation by means of stepwise regression. Kvaal-specific equations for each tooth are shown in Table 2. The table also displays correlation coefficients (*R*), coefficient of determination (*R*^2^) of each equation, and standard error of the estimate in years (SEE). The equation of Teeth 32 performed best among these modules. To consider the comprehensive condition of six teeth, we utilized the module of bimaxillary teeth for the next calculation.

Then, we added the elements of the third molar mineralization (Teeth 28 and 38) to the new equation based on Kvaal’s method of bimaxillary teeth in the training group, which contained the third molar in both upper and lower jaws on the left side. The different stages of third molar quantified by Demirjian’s method were assigned from numbers one to nine, based on the following equation:


$$ \mathrm{Age}=7.124+0.753\ \left(\mathrm{D}28\right)+0.550\ \left(\mathrm{D}38\right)+9.560\ \left(\mathrm{C}\right). $$

*R* ^2^ and SEE values from the regression models of Kvaal-specific equation were 0.053 and 2.008 years, respectively (Table 3). After adding the third molar mineralization of Teeth 28 and 38, the regression models’ *R*^2^ increased to 0.513 and SEE decreased to 1.482 years.

**Table 3:** *R* ^2^ and SEE calculated from the new equation based on Kvaal’s method of left mandibular lateral incisor (32) with and without adding the third molar mineralization regression model.

Regression model	*R* ^2^	SEE
Kvaal-specific equation	0.053	2.008
Combined specific method	0.513	1.482

*R* ^2^, coefficient of determination; SEE, standard error of the estimate in years.

**Table 4:** The statistics of residuals obtained using the specific model of subadults in northern China and other estimation models.

	CA–DA				
Equation	Mean	SD	Min	Max	*P*-value
Kvaal’s method [9]	67.92	8.97	54.85	118.67	0.001^*^
Li’s Chinese specific method [18]	−17.24	4.86	−25.02	−5.88	0.001^*^
Kvaal-specific method (present study)	−1.08	2.12	−4.42	2.76	0.001^*^
Demirjian’s stage method [16]	−1.00	1.68	−4.16	2.87	0.001^*^
Combined specific method (present study)	−0.93	1.35	−4.39	2.22	0.000^*^

SD, standard deviation; *P*-value assessed using *t*-test.

^*^ *P* < 0.05.

Except three methods we have mentioned above (Kvaal’s method, Kvaal-specific method, and combined specific method), two models of age estimation were also added to make a comparison. One was the published method developed by Kvaal’s method specifically used for 20–65 years in northern China [18], and the other was established by the mineralization stages of left maxillary and mandibular third molar [12]. These five methods were verified by the testing group, and the results are shown in Table 4. The combined specific method added with mineralization of third molar had the lowest difference between CA and DA. The differences between CA and DA obtained from the five modules were statistically significant.

## Discussion

We chose to focus on the subadults aged between 15 and 21 years for the following reasons: (i) Demirjian’s method, as an accurate method of estimating DA of teenagers, cannot be used for older individuals [19]; (ii) there are a few accurate methods of DA estimation for our selected age group, and the starting ages of their cohort based on Kvaal’s method were over 20 years old and the age spans were relatively large [18, 20, 21]. Therefore, the selected age range between 15 and 21 years may have an important implication for age estimation.

In 1995, Kvaal et al*.* [9] established a method to estimate DA of a Norwegian population, and the regression equations they created were deemed to be satisfactory (*r*^2^ = 0.76). In 2005, Paewinsky et al*.* [10] used Kvaal’s method to estimate the DA of a German population. They concluded that the width ratios from different levels showed a significant correlation with the CA, and they found the highest coefficient of determination to be in the lateral incisors (*r*^2^ = 0.913). Then, they formulated new regression equations based on width ratios, which were reportedly more accurate than those using Kvaal’s method. However, studies using Kvaal’s method have shown distinct results. Bosmans et al*.* [22] evaluated 197 Belgians aged 19–75 years and reported no significant difference between CA and DA regardless of whether six teeth or three mandibular teeth were included for analyses. Contrary to the results of Bosman et al*.*, Landa et al*.* [15] concluded that Kvaal’s method significantly underestimated the age of Spanish Caucasians (14–60 years, SD = 14.8 years) and that Kvaal’s method was not applicable for this population. Roh et al*.* [20] applied Kvaal’s method to digital OPGs from a Korean population (aged 21–69 years); the age of this population was underestimated by the original equation (SD = 11.58 years). Li et al*.* [18] collected 360 digital panoramic radiographs from individuals in northern China (aged 20–65 years) and reported that Kvaal’s method overestimated the CA of this Chinese population (SD = 11.8 years). Our results about the SD of DA estimation by Kvaal’s method used in the testing group was 8.97 years, which indicated that the reliability of this method for DA estimation was debatable in different regions and races.

The correlation coefficients calculated in our study were generally lower than other studies [18, 20, 21, 23]. After ruling out potential causes of observer bias and techniques of observation and measurement, we believed the reasons may be correlated with the chosen study subjects. The development of teeth is related to race and socio-economic conditions. Kvaal et al*.* [9] selected Norwegian adults as their samples, and people from two races may have differing chewing habits and food culture. The original method has been deemed to be inapplicable to Korean, and Indian, and Turkish subjects [20, 21, 24]. However, the method was not completely unreliable. Kanchan-Talreja et al*.* [21] observed that Kvaal’s method underestimated the DA of Indian subjects (25–77 years) and thought that this method was more applicable to young and middle-aged adults. Similarly, the distribution of residuals of Li et al*.* [18] showed that the method they built was more accurate for subjects aged 35–45 years. While for the samples collected from the same area, we used their published specific Chinese formula to estimated DA in our study group and it showed a better performance than original Kvaal’s method (Table 4).

Regarding whether we want to check the feasibility of Kvaal’s method for subadults in northern China, the study subjects may not represent all Chinese people. Compared with other studies [18, 20, 21, 23], we focused on younger age groups. Our study subjects, aged 15–21 years, of northern China showed significant underestimation by Kvaal’s method. Except for the influence of difference ethnicity and eating habit, it seems that the age range we selected may have influence on the applicability of the original Kvaal’s method in our research. Initially, Kvaal’s method was designed for adults aged over 20 years old, while the age group in our research was 15–21 years. The reasons were as follows: (i) it was proved that the difference on length and width of pulp cavity between different age groups (which covered the age group we studied) was statistically significant [25, 26]; (ii) compared with other samples, younger sample may suffer from less physiological defence or physical illness which could affect the morphology of the pulp cavity potentially [18]; and (iii) as described above, for the shortage of method based on developmental stages of third molar, we need to introduce the morphological parameters of Kvaal’s method to increase the accuracy of age estimation. Moreover, a plenty of novel formulas developed by researchers based on Kvaal’s method had wide age range of application, which could result in a large difference between CA and DA in smaller groups [18, 20, 21]. We wanted to narrow the study range and research the accuracy of the original and derivation methods for a more convincing conclusion.

During the statistical analysis, majority of the researchers found that the ratios of length showed lower correlation to ratios of width. The length value implies little contribution to teeth change, and we can infer that the attrition of teeth may imply little contribution to teeth change by age. These results may provide meaningful information for Kvaal’s method in future studies. For example, Li et al*.* [27] found that measured data of maxillary canines were more correlated with CA compared with other teeth in the age estimation of northern Chinese population. This study was inspired by their previous research, which found that the mandibular canines had a relatively high correlation with CA [18]. It suggested that there may be more potential parameters in teeth associated with CA*.*

The accuracy of age estimation by stages of third molar has been studied by researchers, with the age range similar to ours. Liu et al*.* [12] established a system of formulae based on the third molar mineralization in China with good results. The age range of their models was consistent with our study population, and we chose to use their formula for molars (Teeth 28 and 38), as the teeth we observed were on the left side. The results showed that the new equation performs better between 17 and 18 years, and the standard deviation is much lower than that of the third molar mineralization based on Demirjian’s method. Compared to Kvaal-specific models based on the original method, models combined with the ratio C and the third molar mineralization of Teeth 28 and 38 decreased the SEE age from 2.008 years to 1.482 years. These results reflected that the morphological changes of teeth could be used as a parameter to predict DA. Thevissen et al*.* [14] improved Demirjian’s method by combing the morphological parameters of Kvaal’s method. We build an age estimation method based on Demirjian’s method, and the mean difference between CA and DA was −1.00 years. When the mineralization of the third molar was introduced to age estimation, the mean difference between CA and DA was decreased to −0.93 years. The results indicated the accuracy of DA estimation was increased in this age group of northern China, but the weight of parameters for each method in age prediction were relatively lower and needed to be further investigated.

Kvaal et al*.* [9] used periapical radiographs, but we chose OPG to measure the value of teeth to decrease the radiation dose and exposure to patients, and OPG is easier to obtain than periapical radiograph. The use of periapical radiographs may be associated with problems, such as unequal magnification and distortion related to patient positioning; moreover, the superimposition of premolars’ proximal surfaces are also technical flaws of panoramic radiography.

## Conclusion

This study improved the accuracy of Kvaal’s original method for age estimation in subadults in northern China by combing Kvaal’s parameters and mineralization of third molars. Owing to the significant difference between CA and DA, we recommended further research to examine the potential indicators correlated to CA and verified on more OPGs.

## Authors’ contributions

Weifeng Qu and Jifeng Cai contributed to the methodology, formal analysis, and preparation of the manuscript; Bowei Jiang designed the figures; Dan Wen and Wei He contributed to the statistical analysis work; Chudong Wang and Hao Xing collected the samples and rechecked OPGs as the second observer for Kvaal’s method and Demirjian’s stage division method; Zedeng Yang and Jienan Li assisted in the statistical analysis work; Jian Zhou, Ying Liu, and Lagabaiyila Zha designed the study and revised the manuscript. All authors contributed to and approved the final text.

## Compliance with ethical standards

Ethical approval was obtained from the Ethics Committee of Xiangya Hospital of Central South University (No. 2019-S234). Patients’ OPGs were obtained from the Capital Medical University School of Stomatology with written approval for usage in this study.

## Funding

This project was supported by the National Natural Science Foundation of China [NSFC, No. 82002005] and Natural Science Foundation of Hunan Province [2020JJ5787].

## Supplementary Material

Supplementary_tables_owad013
