# Supplementary material for: Combining the third molar mineralization to further improve the accuracy of the Kvaal’s method in dental age estimation of subadults in northern China
Source: Forensic Sci Res. 2023 Mar 28;8(1):24–9. doi: 10.1093/fsr/owad013 (PMC10265960; doi:10.1093/fsr/owad013)
Supplement: Supplementary_tables_owad013 [file supplementary_tables_owad013.docx]

Supplementary Table S1. Distribution of age and sex of samples from training group cohort.

| Age (years) | Sex | | Total |
| --- | --- | --- | --- |
|  | Male | Female |  |
| 15 | 20 | 20 | 40 |
| 16 | 20 | 20 | 40 |
| 17 | 21 | 18 | 39 |
| 18 | 18 | 20 | 38 |
| 19 | 18 | 20 | 38 |
| 20 | 23 | 17 | 40 |
| 21 | 22 | 21 | 43 |
| Total | 142 | 136 | 278 |

Supplementary Table 2. Statistical description of CA and DA predicted by Kvaal’s method.

| Sex | Teeth | Age group | CA |  | DA | SD | Max | Min | CA–DA |
| --- | --- | --- | --- | --- | --- | --- | --- | --- | --- |
|  |  |  | Mean |  | Mean |  |  |  |  |
| Male | 21 | 15.00−15.99 | 15.38 |  | −8.96 | 9.04 | 7.83 | −25.51 | 24.34 |
|  |  | 16.00−16.99 | 16.43 |  | −13.47 | 8.88 | −2.60 | −47.75 | 29.90 |
|  |  | 17.00−17.99 | 17.57 |  | −18.44 | 8.10 | −7.18 | −43.38 | 36.02 |
|  |  | 18.00−18.99 | 18.51 |  | −14.97 | 9.78 | 12.56 | −34.74 | 33.47 |
|  |  | 19.00−19.99 | 19.51 |  | −12.55 | 7.24 | 3.67 | −24.48 | 32.05 |
|  |  | 20.00−20.99 | 20.49 |  | −11.65 | 9.68 | 2.31 | −36.84 | 32.14 |
|  |  | 21.00−21.99 | 21.37 |  | −16.00 | 11.19 | 1.59 | −55.00 | 37.37 |
|  | 22 | 15.00−15.99 | 15.38 |  | −20.15 | 9.32 | −0.87 | −35.69 | 35.53 |
|  |  | 16.00−16.99 | 16.43 |  | −17.68 | 6.13 | −5.11 | −28.25 | 34.10 |
|  |  | 17.00−17.99 | 17.57 |  | −22.55 | 7.75 | −11.83 | −38.57 | 40.13 |
|  |  | 18.00−18.99 | 18.51 |  | −17.82 | 7.75 | −2.57 | −36.15 | 36.32 |
|  |  | 19.00−19.99 | 19.51 |  | −17.31 | 7.52 | 0.26 | −34.02 | 36.81 |
|  |  | 20.00−20.99 | 20.49 |  | −17.67 | 6.83 | −8.88 | −30.44 | 38.16 |
|  |  | 21.00−21.99 | 21.37 |  | −19.65 | 7.88 | −7.29 | −36.09 | 41.02 |
|  | 25 | 15.00−15.99 | 15.38 |  | −44.88 | 10.36 | −26.52 | −72.27 | 60.26 |
|  |  | 16.00−16.99 | 16.43 |  | −39.34 | 10.28 | −16.13 | −66.89 | 55.76 |
|  |  | 17.00−17.99 | 17.57 |  | −46.05 | 7.72 | −26.42 | −60.40 | 63.63 |
|  |  | 18.00−18.99 | 18.51 |  | −43.14 | 7.97 | −31.41 | −59.48 | 61.64 |
|  |  | 19.00−19.99 | 19.51 |  | −44.64 | 13.06 | −23.48 | −79.28 | 64.14 |
|  |  | 20.00−20.99 | 20.49 |  | −42.37 | 12.00 | −17.89 | −69.82 | 62.86 |
|  |  | 21.00−21.99 | 21.37 |  | −43.55 | 11.41 | −25.18 | −65.61 | 64.92 |
|  | 32 | 15.00−15.99 | 15.38 |  | −27.20 | 10.92 | −11.58 | −50.47 | 42.58 |
|  |  | 16.00−16.99 | 16.43 |  | −30.61 | 11.06 | −16.16 | −61.34 | 47.03 |
|  |  | 17.00−17.99 | 17.57 |  | −35.37 | 11.95 | −19.52 | −74.78 | 52.95 |
|  |  | 18.00−18.99 | 18.51 |  | −30.29 | 10.31 | −8.20 | −46.16 | 48.79 |
|  |  | 19.00−19.99 | 19.51 |  | −28.36 | 11.51 | −11.17 | −66.52 | 47.87 |
|  |  | 20.00−20.99 | 20.49 |  | −34.22 | 11.77 | −8.50 | −57.67 | 54.71 |
|  |  | 21.00−21.99 | 21.37 |  | −21.35 | 10.07 | −0.87 | −40.51 | 42.72 |
|  | 33 | 15.00−15.99 | 15.38 |  | −15.57 | 8.88 | −2.39 | −37.54 | 30.95 |
|  |  | 16.00−16.99 | 16.43 |  | −24.50 | 12.48 | −5.83 | −74.68 | 40.92 |
|  |  | 17.00−17.99 | 17.57 |  | −21.35 | 7.62 | −7.78 | −37.07 | 38.92 |
|  |  | 18.00−18.99 | 18.51 |  | −21.30 | 12.38 | −3.81 | −57.16 | 39.80 |
|  |  | 19.00−19.99 | 19.51 |  | −18.97 | 8.45 | −2.65 | −33.28 | 38.48 |
|  |  | 20.00−20.99 | 20.49 |  | −15.88 | 8.13 | −0.47 | −32.26 | 36.36 |
|  |  | 21.00−21.99 | 21.37 |  | −21.43 | 10.48 | 0.56 | −45.11 | 42.81 |
|  | 34 | 15.00−15.99 | 15.38 |  | −7.48 | 37.15 | 28.32 | −167.12 | 22.86 |
|  |  | 16.00−16.99 | 16.43 |  | 1.86 | 10.20 | 19.65 | −27.96 | 14.56 |
|  |  | 17.00−17.99 | 17.57 |  | −3.62 | 14.19 | 11.93 | −37.53 | 21.20 |
|  |  | 18.00−18.99 | 18.51 |  | −3.95 | 13.93 | 18.07 | −35.95 | 22.45 |
|  |  | 19.00−19.99 | 19.51 |  | −4.43 | 12.57 | 12.87 | −29.50 | 23.94 |
|  |  | 20.00−20.99 | 20.49 |  | 0.06 | 15.06 | 21.55 | −35.52 | 20.43 |
|  |  | 21.00−21.99 | 21.37 |  | 1.28 | 10.74 | 18.76 | −25.67 | 20.09 |
| Female |  |  |  |  |  |  |  |  |  |
|  | 21 | 15.00−15.99 | 15.38 |  | −9.34 | 11.40 | 10.32 | −39.81 | 24.72 |
|  |  | 16.00−16.99 | 16.42 |  | −11.55 | 8.65 | 1.67 | −26.64 | 27.97 |
|  |  | 17.00−17.99 | 17.48 |  | −9.18 | 11.38 | 10.09 | −48.37 | 26.65 |
|  |  | 18.00−18.99 | 18.38 |  | −9.03 | 7.97 | 3.66 | −24.74 | 27.41 |
|  |  | 19.00−19.99 | 19.57 |  | −10.73 | 8.55 | 7.86 | −27.98 | 30.29 |
|  |  | 20.00−20.99 | 20.56 |  | −14.46 | 11.56 | 3.35 | −31.87 | 35.01 |
|  |  | 21.00−21.99 | 21.50 |  | −18.55 | 8.88 | −2.06 | −41.36 | 40.05 |
|  | 22 | 15.00−15.99 | 15.38 |  | −19.76 | 8.01 | −0.20 | −34.51 | 35.14 |
|  |  | 16.00−16.99 | 16.42 |  | −17.32 | 9.83 | 2.45 | −36.31 | 33.74 |
|  |  | 17.00−17.99 | 17.48 |  | −14.63 | 6.44 | −3.47 | −29.09 | 32.10 |
|  |  | 18.00−18.99 | 18.38 |  | −14.31 | 6.32 | −3.57 | −25.06 | 32.69 |
|  |  | 19.00−19.99 | 19.57 |  | −21.33 | 15.37 | 0.82 | −76.69 | 40.90 |
|  |  | 20.00−20.99 | 20.56 |  | −21.12 | 11.61 | 0.56 | −42.49 | 41.68 |
|  |  | 21.00−21.99 | 21.50 |  | −22.79 | 12.41 | 1.36 | −43.93 | 44.29 |
|  | 25 | 15.00−15.99 | 15.38 |  | −43.33 | 9.67 | −27.31 | −69.11 | 58.71 |
|  |  | 16.00−16.99 | 16.42 |  | −42.31 | 7.56 | −25.30 | −55.73 | 58.73 |
|  |  | 17.00−17.99 | 17.48 |  | −36.38 | 9.67 | −19.69 | −57.85 | 53.86 |
|  |  | 18.00−18.99 | 18.38 |  | −40.25 | 12.71 | −17.64 | −79.33 | 58.63 |
|  |  | 19.00−19.99 | 19.57 |  | −44.72 | 12.82 | −21.33 | −76.36 | 64.29 |
|  |  | 20.00−20.99 | 20.56 |  | −44.58 | 17.00 | −15.48 | −87.23 | 65.14 |
|  |  | 21.00−21.99 | 21.50 |  | −45.58 | 11.90 | −24.59 | −69.72 | 67.08 |
|  | 32 | 15.00−15.99 | 15.38 |  | −21.35 | 10.07 | −0.87 | −40.51 | 36.73 |
|  |  | 16.00−16.99 | 16.42 |  | −23.96 | 13.16 | 0.67 | −62.40 | 40.38 |
|  |  | 17.00−17.99 | 17.48 |  | −15.11 | 8.98 | −0.52 | −36.29 | 32.58 |
|  |  | 18.00−18.99 | 18.38 |  | −17.30 | 8.36 | −4.40 | −31.48 | 35.68 |
|  |  | 19.00−19.99 | 19.57 |  | −20.38 | 11.04 | −7.00 | −51.38 | 39.95 |
|  |  | 20.00−20.99 | 20.56 |  | −26.22 | 12.38 | −2.67 | −44.16 | 46.77 |
|  |  | 21.00−21.99 | 21.50 |  | −28.96 | 10.96 | −6.00 | −44.96 | 50.46 |
|  | 33 | 15.00−15.99 | 15.38 |  | −17.14 | 7.89 | −2.01 | −35.81 | 32.52 |
|  |  | 16.00−16.99 | 16.42 |  | −16.94 | 9.24 | 0.47 | −32.81 | 33.36 |
|  |  | 17.00−17.99 | 17.48 |  | −10.07 | 5.98 | 0.82 | −26.49 | 27.54 |
|  |  | 18.00−18.99 | 18.38 |  | −10.95 | 8.03 | 3.87 | −33.79 | 29.33 |
|  |  | 19.00−19.99 | 19.57 |  | −25.08 | 46.03 | −1.15 | −241.86 | 44.65 |
|  |  | 20.00−20.99 | 20.56 |  | −16.91 | 8.81 | −1.25 | −33.08 | 37.46 |
|  |  | 21.00−21.99 | 21.50 |  | −20.50 | 11.86 | −2.86 | −59.49 | 42.00 |
|  | 34 | 15.00−15.99 | 15.38 |  | −2.21 | 9.69 | 14.25 | −24.84 | 17.58 |
|  |  | 16.00−16.99 | 16.42 |  | −0.30 | 14.54 | 30.77 | −31.96 | 16.71 |
|  |  | 17.00−17.99 | 17.48 |  | 10.09 | 11.36 | 31.76 | −13.89 | 7.38 |
|  |  | 18.00−18.99 | 18.38 |  | 12.80 | 10.20 | 28.48 | −5.45 | 5.58 |
|  |  | 19.00−19.99 | 19.57 |  | 9.54 | 15.94 | 56.81 | −22.28 | 10.03 |
|  |  | 20.00−20.99 | 20.56 |  | −0.18 | 18.14 | 29.05 | −25.29 | 20.74 |
|  |  | 21.00−21.99 | 21.50 |  | −1.84 | 16.20 | 31.75 | −30.43 | 23.34 |

CA, chronological age; DA, dental age; SD: standard deviation; Max: maximum dental age; Min: minimum dental age; CA–DA= mean CA–mean DA.
